# Supplementary material for: A framework for estimating the determinants of spatial and temporal variation in vital rates and inferring the occurrence of unobserved extreme events
Source: R Soc Open Sci. 2018 Mar 7;5(3):171087. doi: 10.1098/rsos.171087 (PMC5882670; doi:10.1098/rsos.171087)
Supplement: Additional tables, figures, and methods [file rsos171087supp1.pdf]

Electronic Supplementary Material

A framework for estimating the determinants of spatial and temporal variation in vital rates and inferring the occurrence of unobserved extreme events

Simone Vincenzi[1*], Dusan Jesensek[2], Alain J Crivelli[3]

[1]Institute of Marine Sciences, University of California, Santa Cruz, CA 95064, simon.vincenz@gmail.com

[2]Tolmin Angling Association, Most na Soci, Slovenia, rdt.val@siol.net

[3]Station Biologique de la Tour du Valat, Le Sambuc, F-13200, Arles, France, a.crivelli@tourduvalat.org

*Corresponding author, simon.vincenz@gmail.com

Table S1. Topological characteristics of Upper Volaja. Stream altitude is between 725 (Sector 4) and 683 (Sector 1) m.

Sector	Length (m)	Area (m^2)	Slope	Number of pools	Pool area (m2)
1	44.52	136.57	13%	5	65.59
2	78.53	254.59	17%	5	88.55
3	47.72	134.57	16%	3	43.46
4	93.87	220.54	15%	4	60.74
Total	264.64	746.27	15% (avg)	17	258.34

Table S2. Symbols and abbreviations used in the main text.

Symbol or Abbreviation	*Explanation*
GDDs	Growing degree-days
D_{0+}	Density of age-0+ fish
$D_{>0+}$	Density of fish older than 0+
L_{0+}, $\bar{L}_{0+}$	Length and mean length of fish at age 0+
vBGF	Von Bertalanffy Growth Function
L_{∞}	Asymptotic size in the vBGF
k	Growth coefficient in the vBGF
t_0	Age at which size is equal to 0 in the vBGF
u	Individual random effect for vBGF k
v	Individual random effect for vBGF L_{∞}
σ_u	Standard deviation of the distribution of individual random effect for vBGF k
σ_v	Standard deviation of the distribution of individual random effect for vBGF L_{∞}
x_{ij}	Continuous predictor of vBGF's parameters for individual i in group j
ε_{ij}	Error term of the vBGF for individual i in group j

σ_{ε}^{2}	Variance of the error term for the vBGF
α, β	Categorical predictors of vBGF's parameters for individual *i* in group *j*
$D_{>0+,born}$	Density of fish older than 0+ when the fish/cohort was born
$D_{>0+,m}$	Mean of $D_{>0+}$ at year *t* in September and *t*+1 in June
Cohort	Year-class
$Data_W$	Whole dataset
$Data_S$	Dataset including (a) trout that were sampled once at age 1+, and (b) trout that were sampled multiple times in the same sector and were sampled for the first time at age 1+
$Data_D$	Dataset including all cohorts for which density and water temperature in the first year of life are known
G_d	Daily growth in size
Season	Sampling season: June-September (*Summer*), September-June (*Winter*)
$D_{s,t}$	Density of spawners at yeat *t*
R_t	Recruitment (density of age-0+ in September) at year *t*
GAM	Generalized Additive Model
GAMM	Generalized Additive Mixed-Model
$\bar{T}$	Mean temperature during a *Season*
Time	Sampling occasion in the model of survival
ϕ	Apparent survival of tagged fish
p	Probability of capture in the model of survival

CJS	Cormack-Jolly-Seber model of survival
σ_{0+}	Apparent survival of fish from age 0+ to age 1+

Table S3. Estimates of number and density of fish alive, and probability of capture in each *Year* and *Month* for fish aged 0+ (0) or 1+ and older (1). P_Est, P_Se = point estimate and standard error of probability of capture; N_Obs = number of fish sampled; N_Est, N_LCI, N_UCI = point estimate and lower and upper 95% CI of number of fish. D_Est, D_LCI, D_UCI = point estimate and lower and upper 95% CI of density of fish (fish ha^{-1}). There was complete recruitment failure in 2014.

Year	Month	P_Est	P_Se	N_Obs	N_Est	N_LCI	N_UCI	D_Est	D_LCI	D_UCI	Age
2004	Sept	0.92	0.04	59	59	57.62	60.38	790.60	772.11	809.09	0
2005	Sept	0.90	0.05	60	60	58.03	61.97	804.00	777.64	830.36	0
2006	Sept	0.94	0.05	30	30	29.24	30.76	402.00	391.79	412.21	0
2007	Sept	0.88	0.06	38	38	36.21	39.79	509.20	485.26	533.14	0
2008	Sept	0.72	0.12	31	33	27.19	38.81	442.20	364.41	519.99	0
2009	Sept	0.94	0.07	15	15	14.46	15.54	201.00	193.78	208.22	0
2010	Sept	0.80	0.19	8	8	6.30	9.70	107.20	84.46	129.94	0
2011	Sept	0.81	0.13	17	17	14.69	19.31	227.80	196.90	258.70	0
2012	Sept	0.88	0.05	53	53	50.88	55.12	710.20	681.81	738.59	0
2013	Sept	0.77	0.13	23	24	20.29	27.71	321.60	271.93	371.27	0
2014	Sept	0.00	0.00	0	0	0.00	0.00	0.00	0.00	0.00	0
2015	Sept	0.86	0.05	65	65	62.17	67.83	871.00	833.10	908.90	0
2004	Sept	0.90	0.01	543	548	542.40	553.60	7343.20	7268.13	7418.27	1
2005	June	0.90	0.02	464	468	462.85	473.15	6271.20	6202.23	6340.17	1
2005	Sept	0.95	0.01	502	503	500.61	505.39	6740.20	6708.15	6772.25	1
2006	June	0.91	0.02	385	388	383.71	392.29	5199.20	5141.73	5256.67	1

2006	Sept	0.94	0.01	373	374	371.29	376.71	5011.60	4975.35	5047.85	1
2007	June	0.92	0.02	388	390	386.55	393.45	5226.00	5179.83	5272.17	1
2007	Sept	0.94	0.01	390	391	388.14	393.86	5239.40	5201.04	5277.76	1
2008	June	0.89	0.02	409	413	407.69	418.31	5534.20	5463.01	5605.39	1
2008	Sept	0.92	0.01	420	422	418.26	425.74	5654.80	5604.75	5704.85	1
2009	June	0.86	0.02	329	335	328.35	341.65	4489.00	4399.96	4578.04	1
2009	Sept	0.91	0.02	363	366	361.81	370.19	4904.40	4848.21	4960.59	1
2010	June	0.94	0.01	399	400	397.17	402.83	5360.00	5322.13	5397.87	1
2010	Sept	0.90	0.02	334	337	332.72	341.28	4515.80	4458.51	4573.09	1
2011	June	0.90	0.02	320	323	318.48	327.52	4328.20	4267.64	4388.76	1
2011	Sept	0.92	0.02	369	371	367.45	374.55	4971.40	4923.88	5018.92	1
2012	June	0.90	0.02	323	326	321.64	330.36	4368.40	4309.95	4426.85	1
2012	Sept	0.92	0.02	355	357	353.50	360.50	4783.80	4736.84	4830.76	1
2013	June	0.91	0.02	376	379	374.65	383.35	5078.60	5020.32	5136.88	1
2013	Sept	0.90	0.02	335	338	333.60	342.40	4529.20	4470.27	4588.13	1
2014	June	0.90	0.02	415	419	414.08	423.92	5614.60	5548.74	5680.46	1
2014	Sept	0.91	0.02	432	435	430.43	439.57	5829.00	5767.79	5890.21	1
2015	June	0.95	0.01	331	331	329.08	332.92	4435.40	4409.63	4461.17	1
2015	Sept	0.91	0.02	325	327	323.31	330.69	4381.80	4332.37	4431.23	1

Table S4. Proportion of "late incomers" present in the population each year in September. We applied the same ratio of "late incomers" to total number of fish found for cohorts born after the start of sampling to cohorts born before the start of sampling (from 2000 to 2003). Early.inc = "early incomers", i.e. fish that were either born in Upper Volaja or came into Upper Volaja before age 1+ in September. FP_Coh = number of fish from cohorts born before the start of sampling. N.tot = total number of fish aged 1+ or older sampled each September. Late.inc = "late incomers", i.e. fish that were born in AW and came into Upper Volaja when 1+ in September or older. Prop.late.inc = proportion of "late incomers" in Upper Volaja each year in September.

Year	Early.inc	FP_Coh	N.tot	Late.inc	Prop.late.inc
2010	198	13	334	128	0.38
2011	216	8	369	148	0.40
2012	208	6	355	143	0.40
2013	226	5	335	106	0.32
2014	293	6	432	135	0.31
2015	225	1	325	99	0.30

Table S5. Predictors of vBGF parameters L_∞ and k (*Constant* = no predictors except for individual random effects), number of parameters, and AIC of the tested growth models (dataset $Data_W$; only September data).

L_∞	*k*	*npar*	*AIC*
Cohort	Cohort	32	40608.0
Constant	Cohort	19	40736.2
Cohort	Constant	19	40831.2
Constant	Constant	6	41687.4

Table S6. Cohort-specific vBGF models. Linf_est, Linf_lcl, Linf_ucl = point estimate, lower and upper 95% CI of asymptotic size; k_est, k_lcl, k_ucl = point estimate, lower and upper 95% CI of growth coefficient; to_est, t0_lcl, t0_ucl = point estimate, lower and upper 95% CI of time at length zero; DP = data points; P_L1, P_L2, P_L3 = predicted average size at age 1+, 2+, 3+ in September; O_L1, O_L2, O_L3 = observed average size at age 1+, 2+, 3+ in September. NA means data not available. Avg is for parameters and predictions for the model with no predictors (and observations for all brown trout in the dataset).

Cohort	Linf_est	Linf _lcl	Linf _ucl	k_est	k_lcl	k_ucl	t_0_est	t_0_lcl	t_0_ucl	DP	P_L1	P_L2	P_L3	O_L1	O_L2	O_L3
C00	279.46	241.27	317.65	0.51	0.05	0.98	-1.61	-1.72	-1.51	10	206.37	235.71	253.27	NA	NA	NA
C01	246.44	234.25	258.63	0.60	0.43	0.78	-1.61	-1.72	-1.51	58	195.73	218.74	231.32	NA	NA	232.79
C02	228.55	223.00	234.10	0.50	0.45	0.54	-1.61	-1.72	-1.51	382	165.91	190.37	205.29	NA	191.17	203.45
C03	240.28	234.26	246.30	0.33	0.30	0.35	-1.61	-1.72	-1.51	721	137.80	166.31	186.89	137.56	164.39	184.74
C04	227.04	221.41	232.67	0.34	0.32	0.37	-1.61	-1.72	-1.51	501	134.15	161.05	180.17	131.20	162.88	183.16
C05	227.11	221.55	232.67	0.33	0.30	0.35	-1.61	-1.72	-1.51	548	130.35	157.30	176.75	130.10	158.45	177.78
C06	229.34	223.62	235.06	0.32	0.30	0.34	-1.61	-1.72	-1.51	461	129.53	156.74	176.53	130.54	155.82	174.78
C07	226.24	219.47	233.01	0.32	0.29	0.34	-1.61	-1.72	-1.51	322	127.98	154.83	174.34	126.97	156.38	172.34
C08	225.68	218.99	232.37	0.32	0.30	0.35	-1.61	-1.72	-1.51	353	128.61	155.40	174.79	128.95	153.12	172.64
C09	236.13	228.22	244.04	0.29	0.27	0.32	-1.61	-1.72	-1.51	336	126.87	154.78	175.55	125.12	155.80	176.32
C10	252.69	240.26	265.12	0.27	0.24	0.30	-1.61	-1.72	-1.51	258	126.86	156.32	178.89	126.35	154.69	178.09
C11	244.56	229.16	259.96	0.30	0.26	0.34	-1.61	-1.72	-1.51	153	132.05	160.97	182.45	128.38	160.89	185.60
C12	314.89	282.84	346.94	0.21	0.17	0.24	-1.61	-1.72	-1.51	277	130.89	165.08	192.92	128.87	168.43	192.29
C13	258.97	235.52	282.42	0.29	0.24	0.34	-1.61	-1.72	-1.51	210	137.58	168.13	191.00	136.31	170.46	NA
Avg	224.87	222.21	227.53	0.41	0.39	0.43	-1.25	-1.34	-1.17	4590	134.99	165.06	185.07	131.98	165.91	184.49

Table S7. Sector-specific vBGF models. Linf_est, Linf_lcl, Linf_ucl = point estimate, lower and upper 95% CI of asymptotic size; k_est, k_lcl, k_ucl = point estimate, lower and upper 95% CI of growth coefficient; t_0_est, t_0_lcl, t_0_ucl = point estimate, lower and upper 95% CI of time at length zero; DP = data points; P_L1, P_L2, P_L3 = predicted average size at age 1+, 2+, 3+ in September; O_L1, O_L2, O_L3 = observed average size at age 1+, 2+, 3+ in September.

Sector	Linf_est	Linf _lcl	Linf _ucl	k_est	k_lcl	k_ucl	t_0_est	t_0_lcl	t_0_ucl	DP	P_L1	P_L2	P_L3	O_L1	O_L2	O_L3
1	213.29	204.93	221.65	0.41	0.37	0.46	-1.27	-1.42	-1.11	328	129.69	158.00	176.73	130.53	157.59	175.70
2	213.10	205.55	220.65	0.42	0.37	0.47	-1.27	-1.42	-1.11	611	130.81	159.03	177.58	131.03	158.67	177.91
3	220.14	209.58	230.70	0.40	0.35	0.46	-1.27	-1.42	-1.11	322	132.09	161.38	180.93	132.51	161.42	181.00
4	230.64	220.38	240.90	0.39	0.34	0.44	-1.27	-1.42	-1.11	378	134.82	165.62	186.52	134.99	166.85	184.58

Table S8. Recapture models for the "global model" of probability of survival ϕ(*Cohort* * *Season*). The best recapture model was *p*(*Time*).

Model	npar	AIC	ΔAIC	weight	neg2lnl
ϕ(*Cohort* * *Season*) *p* (~*Time*)	52	13520.62	0	0.74	13416.62
ϕ (*Cohort* * *Season*) *p* (~*Season* + *Time*)	53	13522.74	2.12	0.26	13416.74
ϕ (*Cohort* * *Season*) *p* (~*Season* * *Time*)	63	13542.74	22.12	1.17E-05	13416.74
ϕ (*Cohort* * *Season*) *p* (~*Season*)	32	13594.99	74.36	5.28E-17	13530.99
ϕ (*Cohort* * *Season*) *p* (~bs(*Age*))	34	13597.31	76.68	1.65E-17	13529.31
ϕ (*Cohort* * *Season*) *p* (~*Age*)	32	13600.42	79.79	3.50E-18	13536.41
ϕ (*Cohort* * *Season*) *p* (~1)	31	13605.27	84.65	3.08E-19	13543.27
ϕ (*Cohort* * *Season*) *p* (~*Cohort*)	45	13608.68	88.06	5.60E-20	13518.68

Figure S1

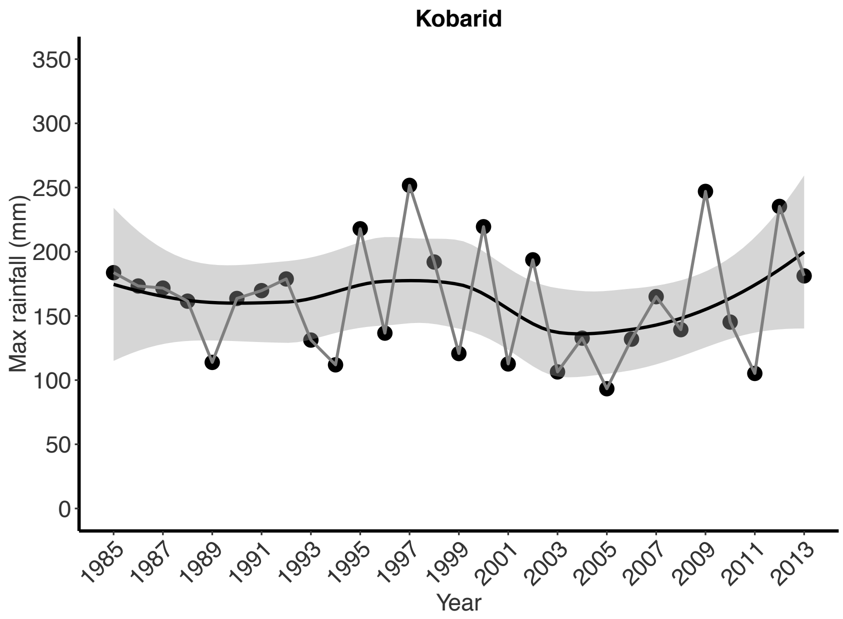

Fig. S1. Maximum annual rainfall recorded in the rainfall station closest to Upper Volaja (Kobarid) along with loess smoothing (gray area delimits 95% CI). Maximum daily rainfall was recorded on November 7th 1997. Daily rainfall similar to that of 1997 was recorded on December 25th 2009 (247 mm)

Figure S2

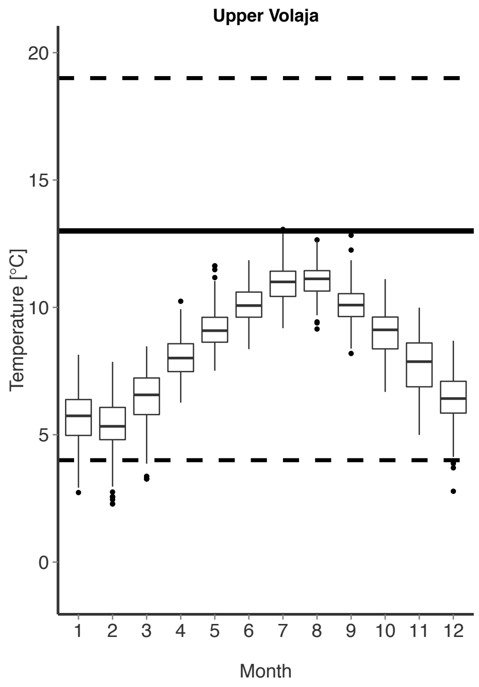

Fig. S2 Boxplots of water temperature recorded (by month) in Upper Volaja between 2004 and 2014. Dashed lines enclose the range of temperatures allowing growth and the thick solid line identifies the temperature for maximum growth according to Elliott et al. (1995) [1].

Figure S3

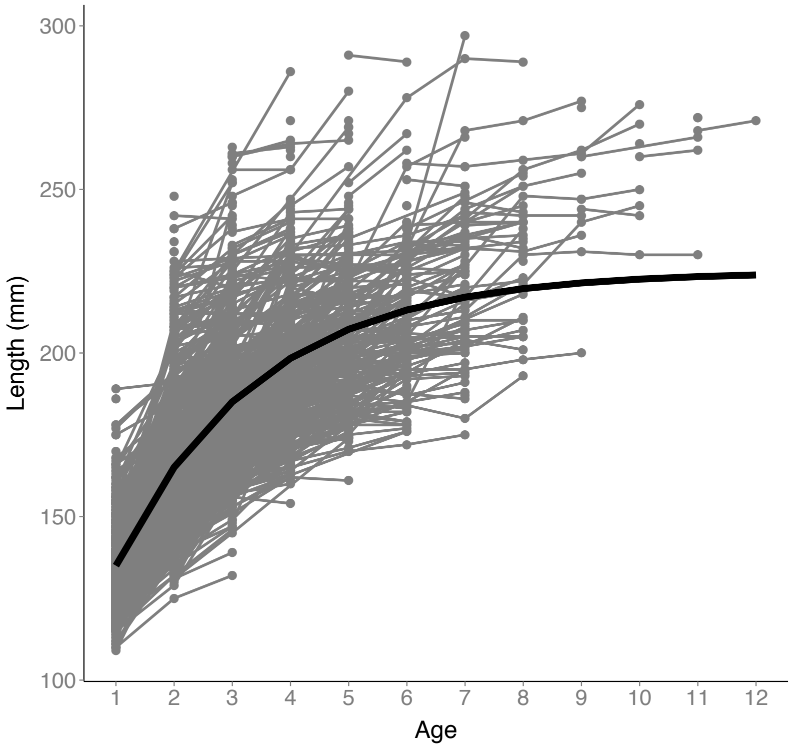

Fig. S3. Individual growth trajectories of brown trout and prediction of the growth model of growth trajectory of the average fish in Eq. (2) in the main text (see Avg in Table ESM 6).

Figure S4

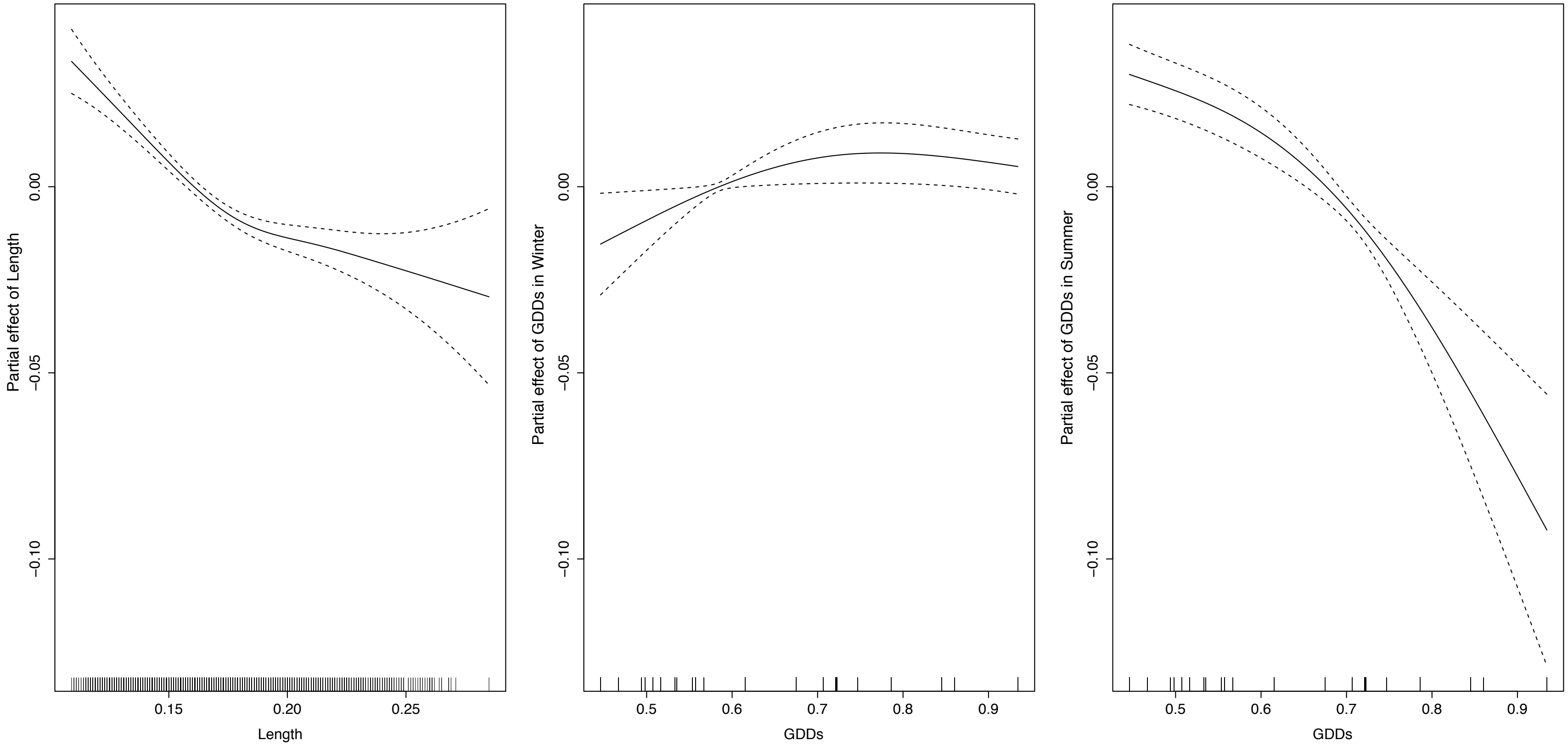

Fig. S4. Partial effects on growth between sampling occasions (mm day^{-1}) of *L* and *GDDs*-by-Season as predicted by the best GAMM model.

Figure S5

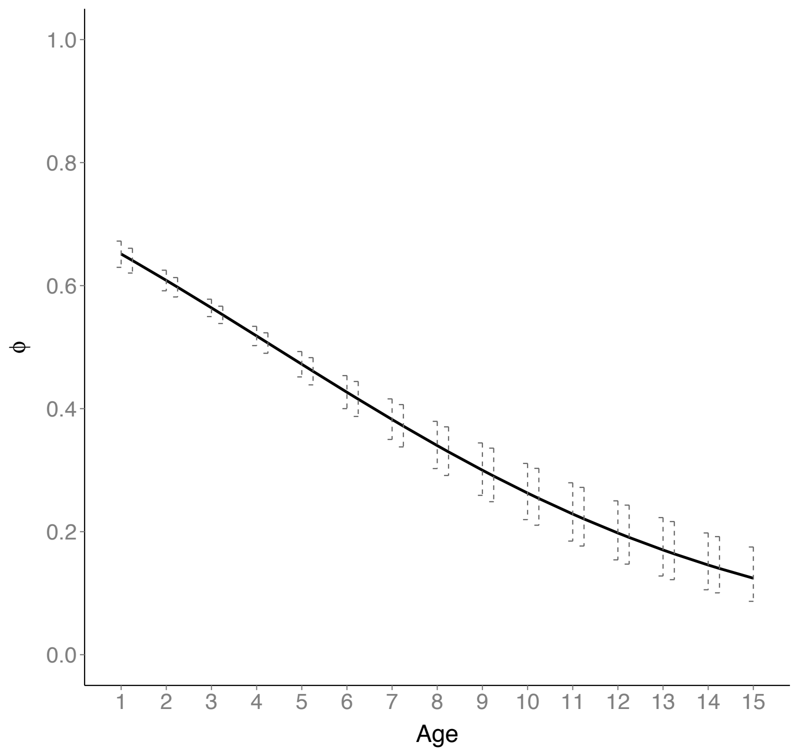

Fig. S5. Point estimates of probability of survival and 95% CI as a non-linear function of *Age* in the population of Upper Volaja (fish are aged 1 in June and 1.25 in September of the second year and so on).

Text S1

When a fish is sampled in September (1+ and older), the following occurrences are possible:

(1) Fish was in Upper Volaja when 0+ (fish born in the stream)

- 1.a fish was sampled when 0+ and the adipose (ad) fin was cut (+)
- 1.b fish was present and not sampled when 0+ (ad not cut)
    - 1.b.1 fish was sampled at 1+ in June, smaller than 115 mm, and the ad was cut (+)
    - 1.b.2 fish was sampled at 1+ in June, bigger than 115 mm (thus getting tagged), and the ad was not cut (-)
    - 1.b.3 fish was not sampled at 1+ in June (missed in two occasions) and the ad was not cut (-)

(2) Fish was in Upper Volaja when 1+ in June but not when 0+

- 2.a fish was sampled when 1+ in June, smaller than 115 mm, and the ad was cut. (+)
- 2.b fish was sampled when 1+ in June, bigger than 115 mm (thus getting tagged), and the ad was not cut. (-)
- 2.c fish was not sampled when 1+ in June (ad was not cut).

(3) Fish was in Upper Volaja when 1+ in September or older, but not when 0+ or 1+ in June.

The two-pass removal and the estimation of capture rates of tagged fish indicate that the probability of sampling a fish when alive and present is between 0.85 and 0.90. Given the very high probability of capturing a fish when present (the point estimate for 0+ is the total number of fish aged 0+ sampled for almost all years) and mortality between sampling occasions, we assumed that 1.b was negligible (see Table ESM 3 for the estimates of density). Since 1+ in June

with $L < 115$ had the ad cut and ~43% of 1+ in June had $L < 115$, we cannot tease apart fish that were sampled when 0+ and fish that were sampled when 1+ in June and had $L < 115$ (we cannot tease apart 1.a from 2.a). Therefore, we have to assign fish to "early incoming" group (which includes fish born in the stream and fish migrating from AW when at max aged 1+ in June, that is 1.a + 2.a + 2.b) and "late incoming" group (fish that migrate into the streams when older than age 1+ in June, that is (3)).

Given the high capture rates, we also assumed that 2.c. was negligible (it will tend to slightly increase the proportion of "early incomers").

Text S2

When using shared *k* and L_∞ among all fish in a population (i.e. no predictors for either *k* or L_∞) or when using *Cohort* as predictor, we used the complete tag-recapture dataset for Upper Volaja (datasets $Data_W$: *n* of individuals = 2414, *n* of length data = 4590).

Some fish were born before both density and temperature data were available. For instance, the oldest fish sampled in Upper Volaja was born in 2000, but $D_{>0+,born}$ and water temperature were first estimated and recorded in 2004. Since we wanted to compare the explanatory power of the growth models when $D_{>0+,born}$, *GDDs*, *Cohort,* were used as predictors of vBGF's parameters, we used a subset of the whole datasets (datasets $Data_D$: *n* of individuals = 1524, *n* of length data = 2919) when using $D_{>0+,born}$ and *GDDs* as potential predictors. For both analyses, we used AIC to select the best model.

The inclusion of trout captured in different sectors throughout their lifetime would not allow using sampling sector as predictor of vBGF's parameters in the formulation in Eq (2). Thus, when including sampling sector as predictor, we used a subset of $Data_W$ ($Data_S$: *n* of individuals = 912, *n* of length data = 1639) that included (a) trout that were sampled once at age 1+, and (b) trout that were sampled multiple times in the same sector and were sampled for the first time at age 1+ (the latter due to avoid bias introduced by fish coming into Upper Volaja from AW, thus by fish that had grown for years in a different environment). Although we cannot exclude that fish sampled multiple times in the same sampling sector had moved between sectors outside the days of sampling, we assumed that, in case of (b), trout stayed permanently in the same sector. When using sampling sector as predictor of VBGF's parameters, we only analyzed model results without comparing model fit to other model formulations.

Text S3

Our goal was to investigate the effects of mean temperature, early density, season, age, sampling occasion on variation in probability of survival of tagged fish using continuous covariates ($D_{>0+}$, mean temperature between sampling intervals $\bar{T}$) at the same time of categorical predictors (*Cohort, Time*, *Season*).

Two probabilities can be estimated from a capture history matrix: ϕ, the probability of apparent survival, and p, the probability that an individual is captured when alive [2].

We used the Cormack–Jolly–Seber (CJS) model as a starting point for the analyses [2]. The global starting model, that is the model with the maximum parameterization for categorical predictors, was different for each population. For both ϕ and p, a multiplicative interaction between *Cohort* and *Time* (i.e. the interval between two consecutive sampling occasions) was included. We could not use the "true" global model of survival with interaction between *Cohort*, *Time*, and *Season* as the model failed to converge.

We tested the goodness-of-fit of the CJS model with the program Release. The global model was a good starting point to model survival and capture probabilities. All other survival models tested were simplified versions of this global starting model, with the potential addition of covariates. We modeled the seasonal effect (*Season*) as a simplification of full time variation, dividing each year in two periods: June to September (*Summer*) and September to June (*Winter*). Since the length of the two intervals (*Summer* and *Winter*) was different, we estimated probability of survival on a common annual scale.

We only tested models with a potential biological interpretation. Normalized Akaike weights (AIC weights) represent the relative probability of a model being closest to the unknown process

that generated the data [3]. From the global model, we first modeled recapture probability by allowing the recapture probability to vary among *Cohort*, *Time*, and *Season*. We then used the recapture model with the lowest AIC to model survival probabilities. Fixing the recapture probability component of the model allowed the survival component of the model to be compared, as any difference in AIC and AIC weight given to individual models would be due to the survival component [4].

Both *Age* and mean temperature between sampling intervals $\bar{T}$ were introduced as either non-linear (as B-splines, Boor 2001) or linear predictors, while $D_{>0+}$ was introduced only as a linear predictor. In addition, we tested whether, after taking account of *Cohort,* probability of survival of trout that was born in AW was different from that of fish born in Upper Volaja. In this case, we used a subset of the whole dataset that included cohorts born between 2004 and 2010 included.

Unless otherwise noted, in this work probability of survival ϕ refers to an annual temporal scale. We use the symbol σ for probability of survival at the population level. We carried out the analysis of probability of survival using the package *marked* [6] for R. Tag-loss is not taken into account in *marked*, although the large number of samples and low proportion of fish losing their tag (<10%) should only slightly bias downwards the survival estimates.

Text S4

Because fish were not tagged when 0+, we assumed a binomial process for estimating the probability σ_{0+} of first overwinter survival (0+ to 1+). We estimated survival by dividing the number of 0+ with the adipose fin cut in September by the estimated number of 1+ with the adipose fin cut in June. We did not use density of 0+ and 1+ the following year since newborns (and 1+ fish the following year before June sampling) can enter the sampling section after the September sampling, thus inflating the estimate of apparent survival. The estimate of the number of 1+ with the adipose fin cut ($\hat{A}_{dc}$) is thus $\hat{A}_{dc} = \frac{A_{dc}}{p_s}$, where A_{dc} is the sampled number of fish aged 1+ with the adipose fin cut and p_s is the probability of capture at time *t* of fish that were alive at time *t*. We estimated the standard error of the parameter of the binomial distribution using the delta method [7].

Ethics statement

All sampling work was approved by the Ministry of Agriculture, Forestry and Food of Republic of Slovenia and the Fisheries Research Institute of Slovenia. Original title of the Plan: RIBISKO - GOJITVENI NACRT za TOLMINSKI RIBISKI OKOLIS, razen Soce s pritoki od izvira do mosta v Cezsoco in Krnskega jezera, za obdobje 2006–2011. Sampling was supervised by the Tolmin Angling Association (Slovenia).

References

1. Elliott, J. M., Hurley, M. A. & Fryer, R. J. 1995 A new, improved growth model for brown trout, Salmo trutta. *Funct. Ecol.* **9**, 290–298.

2. Thomson, D. L., Cooch, E. G. & Conroy, M. J. 2009 *Modeling Demographic Processes in Marked Populations*. Springer.

3. Burnham, K. P. & Anderson, D. R. 2002 *Model Selection and Multimodel Inference: a Practical Information-Theoretic Approach*. New York: Springer Verlag.

4. McCallum, H. 2000 *Population Parameters: Estimation for Ecological Models*. Oxford: Blackwell Science.

5. Boor, C. de 2001 *A Practical Guide to Splines*. Springer New York. [cited 2015 Jun. 23].

6. Laake, J. L., Johnson, D. S. & Conn, P. B. 2013 marked: An R package for maximum-likelihood and MCMC analysis of capture-recapture data. *Methods Ecol. Evol.* **4**, 885–890. (doi:10.1111/2041-210X.12065)

7. Hilborn, R. & Mangel, M. 1997 *The Ecological Detective*. Princeton University Press. [cited 2014 Jul. 31].

1. Elliott, J. M., Hurley, M. A. & Fryer, R. J. 1995 A new, improved growth model for brown trout, Salmo trutta. *Funct. Ecol.* **9**, 290–298.

2. Thomson, D. L., Cooch, E. G. & Conroy, M. J. 2009 *Modeling Demographic Processes in Marked Populations*. Springer.

3. Burnham, K. P. & Anderson, D. R. 2002 *Model Selection and Multimodel Inference: a Practical Information-Theoretic Approach*. New York: Springer Verlag.

4. McCallum, H. 2000 *Population Parameters: Estimation for Ecological Models*. Oxford:

Blackwell Science.

5. Boor, C. de 2001 *A Practical Guide to Splines*. Springer New York. [cited 2015 Jun. 23].

6. Laake, J. L., Johnson, D. S. & Conn, P. B. 2013 marked: An R package for maximum-likelihood and MCMC analysis of capture-recapture data. *Methods Ecol. Evol.* **4**, 885–890. (doi:10.1111/2041-210X.12065)

7. Hilborn, R. & Mangel, M. 1997 *The Ecological Detective*. Princeton University Press. [cited 2014 Jul. 31].
