# Supplementary material for: A framework for estimating the determinants of spatial and temporal variation in vital rates and inferring the occurrence of unobserved extreme events
Source: R Soc Open Sci. 2018 Mar 7;5(3):171087. doi: 10.1098/rsos.171087 (PMC5882670; doi:10.1098/rsos.171087)
Supplement: Titles and legends [file rsos171087supp2.pdf]

Title and legends of EMS for “A framework for estimating the determinants of spatial and temporal variation in vital rates and inferring the occurrence of unobserved extreme events”

Figure S1
Title: Maximum annual rainfall.
Legend: Maximum annual rainfall recorded in the rainfall station closest to Upper Volaja (Kobarid) along with loess smoothing (gray area delimits 95% CI). Maximum daily rainfall was recorded on November 7th 1997. Daily rainfall similar to that of 1997 was recorded on December 25th 2009 (247 mm)

Figure S2
Title: Boxplots of water temperature.
Legend: Boxplots of water temperature recorded (by month) in Upper Volaja between 2004 and 2014. Dashed lines enclose the range of temperatures allowing growth and the thick solid line identifies the temperature for maximum growth according to Elliott et al. (1995).

Figure S3
Title: Individual growth trajectories.
Legend: Individual growth trajectories of brown trout and prediction of the growth model of growth trajectory of the average fish in Eq. (2) in the main text (see Avg in Table ESM 6).

Figure S4
Title: Partial effects on growth.
Legend: Partial effects on growth between sampling occasions (mm day^{-1}) of *L* and *GDDs*-by-Season as predicted by the best GAMM model.

Figure S5
Title: Probability of survival as a function of *Age*.
Legend: Point estimates of probability of survival and 95% CI as a non-linear function of *Age* in the population of Upper Volaja (fish are aged 1 in June and 1.25 in September of the second year and so on).

Table S1
Title: Topology.
Legend: Topological characteristics of Upper Volaja. Stream altitude is between 725 (Sector 4) and 683 (Sector 1) m.

Table S2
Title: Symbols and abbreviations
Legend: Symbols and abbreviations used in the main text.

Table S3
Title: Number and density estimates.

Legend: Estimates of number and density of fish alive, and probability of capture in each *Year* and *Month* for fish aged 0+ (0) or 1+ and older (1). P_Est, P_Se = point estimate and standard error of probability of capture; N_Obs = number of fish sampled; N_Est, N_LCI, N_UCI = point estimate and lower and upper 95% CI of number of fish. D_Est, D_LCI, D_UCI = point estimate and lower and upper 95% CI of density of fish (fish ha^{-1}). There was complete recruitment failure in 2014.

Table S4
Title: Proportion of "late incomers".
Legend: Proportion of "late incomers" present in the population each year in September. We applied the same ratio of "late incomers" to total number of fish found for cohorts born after the start of sampling to cohorts born before the start of sampling (from 2000 to 2003). Early.inc = "early incomers", i.e. fish that were either born in Upper Volaja or came into Upper Volaja before age 1+ in September. FP_Coh = number of fish from cohorts born before the start of sampling. N.tot = total number of fish aged 1+ or older sampled each Septebmer. Late.inc = "late incomers", i.e. fish that were born in AW and came into Upper Volaja when 1+ in September or older. Prop.late.inc = proportion of "late incomers" in Upper Volaja each year in September.

Table S5
Title: vBGF parameters.
Legend: Predictors of vBGF parameters L_∞ and *k* (*Constant* = no predictors except for individual random effects), number of parameters, and AIC of the tested growth models (dataset $Data_W$; only September data).

Table S6
Title: Cohort-specific vBGF models.
Legend: Cohort-specific vBGF models. Linf_est, Linf_lcl, Linf_ucl = point estimate, lower and upper 95% CI of asymptotic size; k_est, k_lcl, k_ucl = point estimate, lower and upper 95% CI of growth coefficient; to_est, t0_lcl, t0_ucl = point estimate, lower and upper 95% CI of time at length zero; DP = data points; P_L1, P_L2, P_L3 = predicted average size at age 1+, 2+, 3+ in September; O_L1, O_L2, O_L3 = observed average size at age 1+, 2+, 3+ in September. NA means data not available. Avg is for parameters and predictions for the model with no predictors (and observations for all brown trout in the dataset).

Table S7
Title: Sector-specific vBGF models.
Legend: Sector-specific vBGF models. Linf_est, Linf_lcl, Linf_ucl = point estimate, lower and upper 95% CI of asymptotic size; k_est, k_lcl, k_ucl = point estimate, lower and upper 95% CI of growth coefficient; t_0_est, t_0_lcl, t_0_ucl = point estimate, lower and upper 95% CI of time at length zero; DP = data points; P_L1, P_L2, P_L3 = predicted average size at age 1+, 2+, 3+ in September; O_L1, O_L2, O_L3 = observed average size at age 1+, 2+, 3+ in September. NA means data not available.

Table S8
Title: Recapture models.
Legend: Recapture models for the "global model" of probability of survival ϕ(*Cohort* * *Season*). The best recapture model was p(*Time*).

Text S1
Title: Estimating the proportion of "early incomers" and "late incomers"
Legend: Not applicable.

Text S2
Title: Details on estimation of growth models
Legend: Not applicable.

Text S3
Title: Details on survival models for tagged individuals
Legend: Not applicable.

Text S4
Title: Details on survival models for juveniles
Legend: Not applicable.
